# Unified Language for Knowledge Dissemination: The Vascular Ageing Glossary, an Initiative by VascAgeNet

**DOI:** 10.1007/s44200-023-00041-5

**Published:** 2024-01-29

**Authors:** Elisabetta Bianchini, Rachel E. Climie, Christopher Clemens Mayer, Maria Raffaella Martina, Manasi Nandi, Arno Schmidt-Trucksäss, Patrick Segers, Chloe Park, Giacomo Pucci, Dimitrios Terentes-Printzios, Peter H. Charlton, for VascAgeNet

**Affiliations:** 1 Institute of Clinical Physiology (IFC), National Research Council (CNR), Pisa, Italy; 2 Menzies Institute for Medical Research, University of Tasmania, 17 Liverpool St, Hobart, 7000 Australia; 3 Center for Health and Bioresources, Medical Signal Analysis, AIT Austrian Institute of Technology GmbH, Giefinggasse 4, 1210 Vienna, Austria; 4 School of Cancer and Pharmaceutical Science, Faculty of Life Sciences and Medicine, King’s College London, 150 Stamford Street, London, SE19NH UK; 5 Division of Sport and Exercise Medicine, Department of Sport, Exercise and Health, University of Basel, Grosse Allee 6, 4052 Basel, Switzerland; 6 Department of Clinical Research, University of Basel, Basel, Switzerland; 7 Institute of Biomedical Engineering and Technology, Ghent University, C. Heymanslaan 10, 9000 Ghent, Belgium; 8 MRC Unit for Lifelong Health and Ageing at UCL, UCL, London, UK; 9 Unit of Internal Medicine, Department of Medicine and Surgery, Santa Maria Terni Hospital, University of Perugia, Terni, Italy; 10 First Department of Cardiology, National and Kapodistrian University of Athens, Medical School, Hippokration Hospital, Athens, Greece; 11 Strangeways Research Laboratory, Department of Public Health and Primary Care, University of Cambridge, 2 Worts Causeway, Cambridge, CB1 8RN UK

**Keywords:** Vascular ageing, Glossary, Dissemination

## Abstract

**Objectives:**

In general, a terminology shared and agreed by different stakeholders is important to facilitate communication and cooperation. This holds true in the field of vascular ageing for the benefit of global cardiovascular health. The need to promote a common language and understanding across this area was recognised by VascAgeNet, a collaborative network with relevant and diverse expertise in the vascular ageing field, supported by the European Cooperation in Science and Technology. To contribute to the spread of unified terms in the vascular ageing field, a glossary was created by VascAgeNet and this paper describes the systematic process used for its development.

**Methods:**

An initial list of terms and preliminary definitions were collated from the network. A dedicated team was created to design the glossary development process, to facilitate its implementation and to maximise outreach and dissemination. The key steps of the process were to determine: (1) the target audience; (2) a list of priority terms; (3) a template structure for definitions; (4) methods for collecting feedback and (5) the dissemination plan.

**Results:**

An implementation strategy was provided for each key step and shared within the network; main decisions were agreed by all members of the glossary team. Small groups of definitions were released on a regular basis within a pilot phase including 19 terms (status: 05.09.2023) that were published openly at https://vascagenet.eu/official-glossary.

**Conclusions:**

The strategy for creating the first Vascular Ageing Glossary has been successfully designed and developed within VascAgeNet. A pilot phase covering the first publicly available terms was completed. The glossary is a living document, available to the scientific community, which aims to unify the vascular ageing language.

**Supplementary Information:**

The online version contains supplementary material available at 10.1007/s44200-023-00041-5.

## Introduction and Aim

Multidisciplinary collaborations of researchers, clinicians, developers, etc. benefit from a common language and collective understanding of fundamental principles, concepts and techniques in order to strengthen communication among collaborators. Therefore, a unified language for knowledge exchange could improve the readability of scientific outputs and their impact on society [1].

Unambiguous information and correct definitions of scientific and medical terms, combined with effective outreach and communication strategies, can help individuals to make better informed decisions. This awareness has influenced work concerning medical or scientific glossaries. For example, key terms about vaccination terminology were provided recently as a tool to facilitate communication between healthcare professionals and patients or the wider public, particularly with the arrival of COVID-19 [2, 3]. Similarly, in exposure science, terminology was published to harmonise regulatory and scientific information for exposure and risk assessment in different contexts [4].

The need for a common understanding has also reached the multidisciplinary network VascAgeNet [5], where vascular experts within bioengineering, mathematics, physiology and medicine fields, recognised their diverse understanding on terms related to vascular ageing.

Vascular ageing refers to the ageing process of the arterial system and its association with cardiovascular risk [6] and VascAgeNet [5] aims to improve the translation of its assessment from research into clinical practice. Therefore, it was a logical step to create a glossary to improve the communication and readability of scientific knowledge related to this field for the scientific community and the healthcare sector. The Vascular Ageing Glossary was initiated as an ongoing living process to harmonise, refine, and promote terms and concepts: in this paper, we describe the method for constructing the glossary, which is designed to build a common and shared language in this interdisciplinary field.

## Methods

### Strategy Development

VascAgeNet is a European multidisciplinary collaborative network on vascular ageing, including more than 350 members from 40 + countries funded by the European Cooperation in Science and Technology (COST, CA18216), aimed at developing a network able to improve the translation of vascular ageing assessment into practice [7, 8]. VascAgeNet is structured into five working groups (WGs) dedicated to support interdisciplinary collaboration (WG1), to understand the underlying mechanisms of vascular ageing and the mathematical models used for their assessment (WG2), to perform technological breakthroughs (WG3), to harmonise available data and studies for enabling multicentre research initiatives (WG4), to promote a vascular ageing culture (WG5) [7].

These WGs agreed on the need for a vascular ageing glossary to promote a common language across the field. An open and inclusive call within the network led to the identification of an initial list of 118 terms and preliminary definitions.

**Fig. 1:**
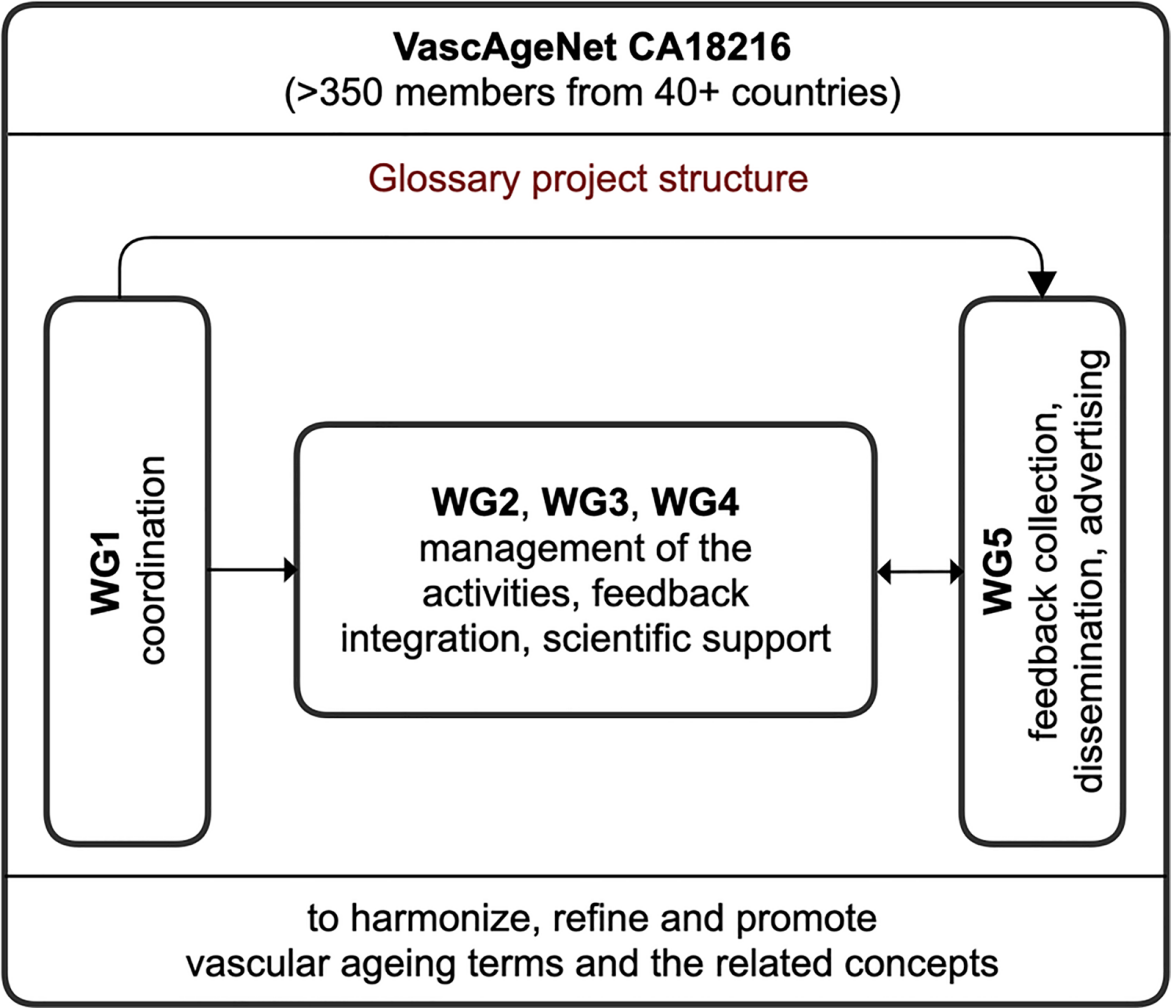
Working groups (WGs) structure and main tasks in the Glossary project

After this first step, a dedicated interdisciplinary glossary team, composed by 7 experts (more than 10 years of experience in the field) including leaders and representatives from all the VascAgeNet WGs (Fig. 1), was created to design and implement a glossary development process [9] to maximise outreach and dissemination. The key steps of the process for managing the creation and maintenance of the glossary were to determine: (1) the target audience; (2) a list of priority terms; (3) a template structure for definitions; (4) methods for collecting feedback and (5) the dissemination plan.

### Pilot Phase

Implementation of a pilot phase was carried out by means of a collaborative and multi-step workplan of the glossary team, based on the development of a subset of definitions, and including the collection of feedback from VascAgeNet members. Fields of expertise within the team included: image and signal processing, vascular ageing cell biology and physiology, bioengineering and clinical application.

A first list composed of 19 terms was developed starting from the initial list and definitions provided by the Network. Selection criteria were based on scientific relevance and expertise within the glossary team; some terms were developed following a call for expression of interest during a VascAgeNet meeting engaging volunteers who supported the co-creation of preliminary definitions based on their expertise. The 19 selected terms were finalised according to a template structure (mentioned in the previous paragraph and detailed in Fig. 3 of the results section). A first review process of the drafted terms was carried out by the glossary team, then feedback in terms of 'accuracy', 'clarity' and 'exhaustiveness' of the definitions was collected from the whole network as described below.

## Results

The development of the glossary project providing the strategy and the pilot phase started in spring 2021 and ended two years later with 19 published terms. The first definitions were released after 7 months from its initiation, small groups of terms were then added every 3–4 months.

### Strategy Development

**Fig. 2:**
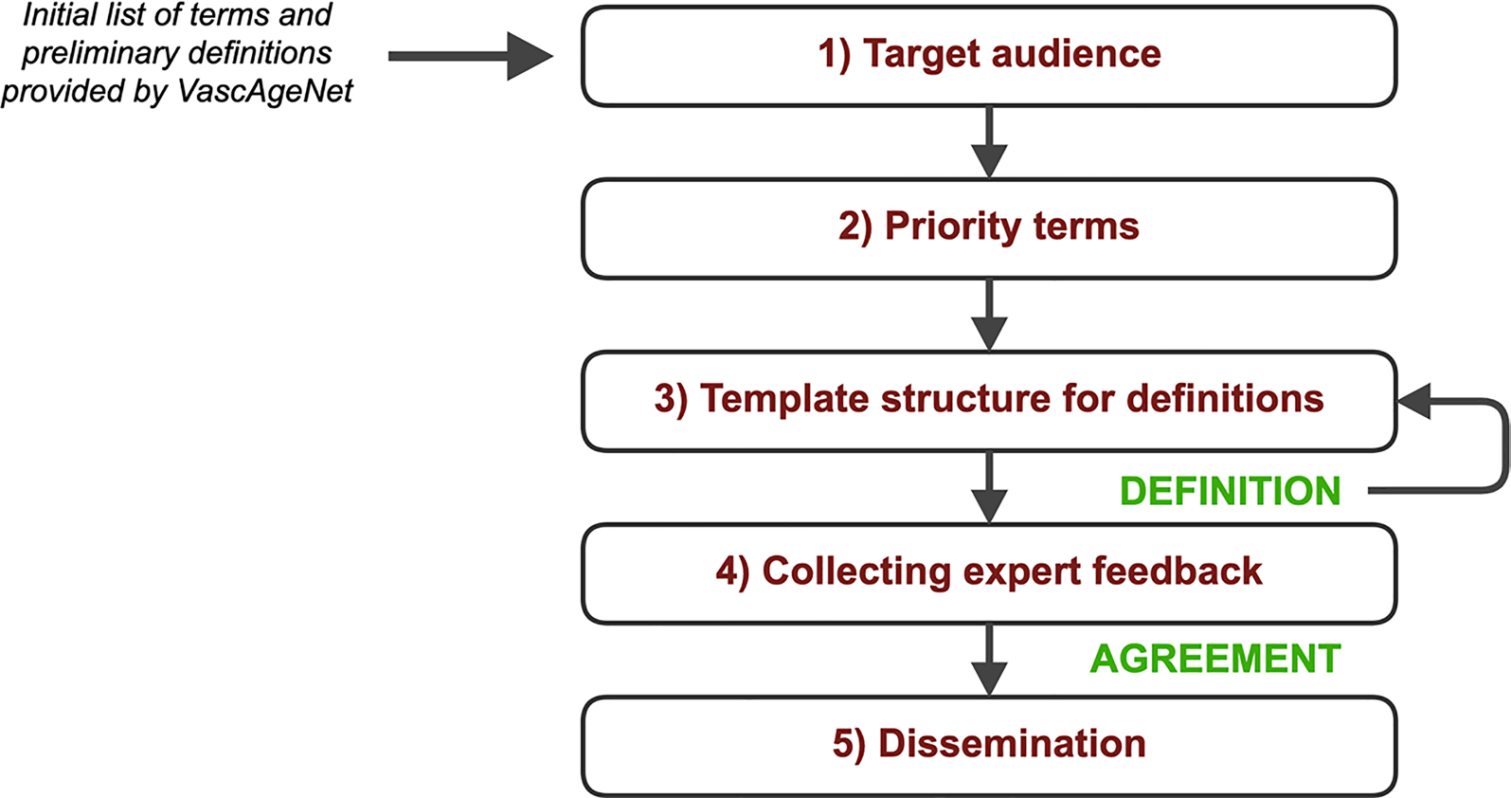
Overview of the key aspects addressed for the glossary development

An implementation strategy was provided by the glossary team for the above-mentioned key aspects (Fig. 2).

**Fig. 3:**
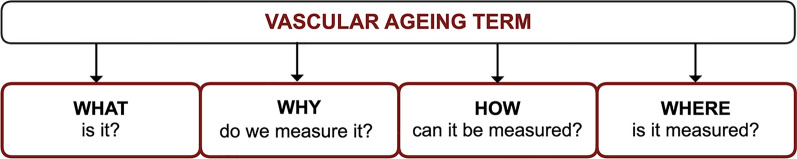
Defined key questions used to draft the terms

More specifically:

1. The target audience was identified as the scientific community, defined as the network of interacting scientists, involved or interested in the vascular ageing field. A dedicated glossary page on the VascAgeNet website was developed.
2. To establish priority terms, the glossary team reviewed the initial list of 118 terms and selected a subgroup of key terms according to their expertise. The glossary terms were divided into 3 main categories: ‘fundamental concepts’, ‘parameters’ and ‘techniques’. The key terms, voted by the network, were mainly parameters and fundamental concepts and answered the key question “How can vascular ageing be measured?”, thus setting the basis for the construction of the glossary.
3. A template structure was designed for definitions with the aim to reach a homogeneous structure facilitating the readability across the glossary. The defined structure was agreed by the glossary team and was based on four questions shown in Fig. 3. Each member of the glossary team finalised specific terms provided by the network according to their field of expertise. If needed, further specific experts within the network were asked to support this phase.

1. A method for collecting and quantifying feedback was developed based on online forms. The glossary development process currently includes two webpages: (1) a password-protected internal webpage of the glossary to collect feedback from VascAgeNet members; (2) a publicly available glossary VascAgeNet webpage (https://vascagenet.eu/official-glossary) where the final version of the terms is accessible. The feedback from the reviewers was collected through the internal webpage and the drafted definitions were subsequently updated. The first stage was a crucial step to improve the drafted terms before open access publication. The review process was quantified in terms of ‘accuracy’, ‘clarity’ and ‘exhaustiveness’. Satisfaction (yes/no) about clarity, accuracy, and level of detail was asked to the reviewers and, where appropriate, any suggestions to improve the terms was allowable through a free text section. After these steps, the final definitions were reviewed and approved by the glossary team, then reported and publicly available on the official glossary VascAgeNet webpage.
2. The dissemination plan was based on the release of small groups of terms on a regular basis on the official website. The dissemination strategy also involves sharing the glossary terms via social media, scientific presentations at international conferences and via this article.

### Pilot Phase

Nineteen peer-reviewed definitions were created as a result of the pilot phase. The published terms were: characteristic impedance, compliance and distensibility coefficient, carotid intima-media thickness, flow mediated dilation, input impedance, pulse wave velocity, wall shear stress, Windkessel model, arterial compliance, applanation tonometry, endothelium, photoplethysmography, pulse wave, pulse pressure amplification, reflection index, reflection magnitude, strain and strain rate, vulnerable plaques and Young’s modulus (two terms are reported as an example in the supplementary materials).

**Fig. 4:**
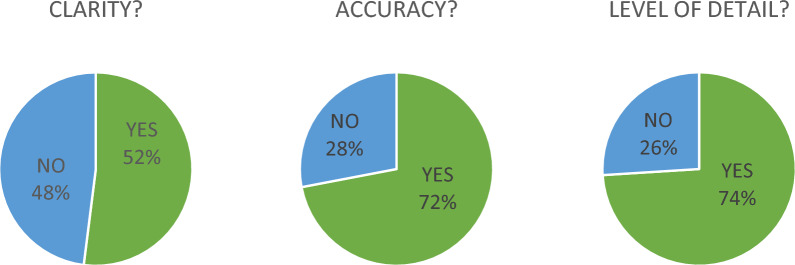
Degree of satisfaction (yes/no) of the reviewers regarding the clarity, accuracy and level of detail of the drafted glossary terms

Around 70 feedbacks were received from the network covering all the terms. The peer-feedback was provided by individuals with diverse expertise, specifically 56% from biomedical engineers, and 41% from clinical medicine and clinical research, 3% from biomedical sciences/non-clinical research or other fields; 56% of reviewers identified themselves as ‘experts’ for the specific terms they were evaluating, 30% identified themselves as a ‘partial experts’. The peer-review process highlighted the need to improve clarity, where initial definitions were rated by 48% of the reviewers as not being clear. In contrast, most reviewers rated the definitions as accurate, and with a good level of detail (Fig. 4).

The reviewers’ suggestions to improve these aspects were evaluated and integrated by the glossary team for the openly published version of the terms. Agreement within the glossary team was considered as final approval for the publication of a term.

## Discussion

The main goal of this project was the development of a tool to promote a shared language in the vascular ageing field including vascular physiology, methodology, technology and medicine similar to those that already exist for mobile applications available for medical terminology, e.g. NIH: Diabetes Glossary (https://apps.apple.com/us/app/nih-diabetes-glossary/id1525266144) or Cardiovascular Medical Terms (https://apps.apple.com/il/app/cardiovascular-medical-terms/id1115127276). We believe that the added value of our initiative lies in the inclusion of expert stakeholders in the glossary team to design the strategy and draft the terms. Each term was completed due to the comprehensive integration of feedback collected from the scientific community. The evaluation of the glossary regarding clarity, accuracy and level of detail by the reviewers was considered a necessary step to ensure quality of the final terms, as reported by literature in this field [10]. In line with the multidisciplinary and translational approach of VascAgeNet, all Working Groups were involved: this proactive collaborative approach was adopted in the design of the glossary as well as in the review process of terminologies and analysis of the feedback.

The development of tools such as this glossary is particularly useful in science and medicine to facilitate the translation of scientific outputs between different stakeholders. Strengthening communication between clinicians, researchers and developers accelerates the innovation process and the development of technologies, improves readability of scientific outputs and their impact on the society [1].

The concept of vascular ageing is strongly related to the risk of developing cardiovascular diseases [11]. The need to promote and improve the assessment of vascular ageing in clinical practice is evident [12]. Our Vascular Ageing Glossary, available to the community, supports the harmonisation of the vascular ageing language to facilitate knowledge’s exchange in the field for the benefit of people.

In regard to the sustainability of the initiative, the glossary team agreed on the need for keeping a dedicated group that meets regularly with specific tasks, to drive continuity and implementation in a possible future phase 2. The team recognised and emphasised the added value of a collective approach based on expertise in the field of vascular ageing. The Vascular Ageing Glossary, presented in the pilot phase, is intended as a starting point. The initiative might be extended through the involvement of international Societies operating in the vascular ageing field, such as the Artery Society (https://www.arterysociety.org). In line with the reported pilot phase, a sustainability strategy of this project might be based on a dedicated expert team managing periodic release of new terms and definitions flanked by social media advertisement and publications concerning glossary updates. In parallel, a funding search could be initiated to support ongoing and future activities. An example might be the development of a mobile app with the two-fold aim to facilitate users' access to published terms and to expand the target audience to the general public. This approach might provide simplified definitions accessible through free mobile tools. Dissemination material and educational videos, already developed by VascAgeNet Working Group 5 who is responsible for education and dissemination, would be integrated in the mobile app. Further app extensions could be based on the integration with advanced supplementary modules to test user’s training after adoption of the system. This project will be fully integrated and promoted within the vascular ageing promoting initiatives in the field.

It is worth noting that some operative improvements might be implemented. For example, it has been suggested that the review process might be simplified, e.g. using an online platform to share drafted terms more easily, thus enhancing interaction and exchange of comments among users.

## Conclusions

The strategy for creating the first Vascular Ageing Glossary has been successfully designed and developed within VascAgeNet. A pilot phase covering the first publicly available terms was completed. The proposed initiative provides an evolving document, available to the scientific community. The glossary will likely support the harmonisation of the vascular ageing language. Furthermore, it is possible that shared vascular ageing terminology may increase the broader societal impact of scientific material related to the field.

### Supplementary Information

Below is the link to the electronic supplementary material.

Supplementary file1 (DOCX 1090 KB)

## Data Availability

The data that support this study are available from the corresponding author upon reasonable request.

## References

[1] Chute CG, Cohn SP, Campbell JR. A framework for comprehensive health terminology systems in the United States: development guidelines, criteria for selection, and public policy implications. J Am Med Inform Assoc. 1998;5:503–10. 10.1136/jamia.1998.0050503. 9824798 10.1136/jamia.1998.0050503 PMC61331

[2] Burke PF, Masters D, Massey G. Enablers and barriers to COVID-19 vaccine uptake: an international study of perceptions and intentions. Vaccine. 2021;39:5116–28. 10.1016/j.vaccine.2021.07.056. 34340856 10.1016/j.vaccine.2021.07.056 PMC8299222

[3] Brennan OC, Moore JE, Moore PJA, Millar BC. Vaccination terminology: a revised glossary of key terms including lay person’s definitions. J Clin Pharm Ther. 2022;47:369–82. 10.1111/jcpt.13516. 34463972 10.1111/jcpt.13516 PMC8656271

[4] Heinemeyer G, Connolly A, von Goetz N, Bessems J, Bruinen de Bruin Y, Coggins MA, Fantke P, Galea KS, Gerding J, Hader JD, Heussen H, Kephalopoulos S, McCourt J, Scheepers PTJ, Schlueter U, van Tongeren M, Viegas S, Zare Jeddi M, Vermeire T. Towards further harmonization of a glossary for exposure science—an ISES Europe statement. J Expo Sci Environ Epidemiol. 2022;32:526–9. 10.1038/s41370-021-00390-w. 34728760 10.1038/s41370-021-00390-w PMC9349032

[5] The COST Action VascAgeNet. Eur Heart J. 2020;41:345. 10.1093/eurheartj/ehz970. 10.1093/eurheartj/ehz970 31942992

[6] Climie RE, Alastruey J, Mayer CC, Schwarz A, Laucyte-Cibulskiene A, Voicehovska J, Bianchini E, Bruno RM, Charlton P, Grillo A, Guala A, Hallab M, Hametner B, Jankowski P, Königsten K, Lebedeva A, Mozos I, Pucci G, Puzantian H, Terentes-Printzios D, Yetik-Anacak G, Park C, Nilsson PM, Weber T. Vascular ageing—moving from Bench towards bedside. Eur J Prev Cardiol. 2023. 10.1093/eurjpc/zwad028. 36738307 10.1093/eurjpc/zwad028 PMC7614971

[7] Climie RE, Mayer CC, Bruno RM, Hametner B. Addressing the unmet needs of measuring vascular ageing in clinical practice-european cooperation in science and technology action vascagenet. Artery Res. 2020;26:71–5. 10.2991/artres.k.200328.001. 10.2991/artres.k.200328.001

[8] Mayer CC, Climie RE, Hametner B, Bruno RM. The european cost action VascAgeNet fostering innovation—when industry comes to science. Artery Res. 2020;26:125–9. 10.2991/ARTRES.K.200430.001. 10.2991/ARTRES.K.200430.001

[9] Velardi P, Poler R, Tomás JV. Methodology for the definition of a glossary in a Collaborative Research Project and its application to a European Network of Excellence. Interoperability Enterp Softw Appl. 2006. 10.1007/1-84628-152-0_28. 10.1007/1-84628-152-0_28

[10] Smith BJ, Tang KC, Nutbeam D. WHO health promotion glossary: new terms. Health Promot Int. 2006;21:340–5. 10.1093/heapro/dal033. 16963461 10.1093/heapro/dal033

[11] Weber T, Wassertheurer S, Hametner B, Mayer CC, Moebus S, Schramm S, Roggenbuck U, Lehmann N, Joeckel KH, Erbel R. Heinz Nixdorf Recall Study, Additive prognostic value of vascular aging and coronary artery calcium for all-cause mortality in the Heinz Nixdorf Recall Study. Eur Heart J. 2020;41:ehaa946.2824. 10.1093/ehjci/ehaa946.2824. 10.1093/ehjci/ehaa946.2824

[12] Panayiotou AG, Park C, Climie RE, Mayer CC, Pucci G, Bianchini E, Weber T, Triantafyllou A. Limitations to implementation of measuring vascular ageing in routine clinical practice. J Hypertens. 2023;41:1054–6. 10.1097/HJH.0000000000003393. 37139698 10.1097/HJH.0000000000003393

