# Supplementary material for: Unified Language for Knowledge Dissemination: The Vascular Ageing Glossary, an Initiative by VascAgeNet
Source: Artery Res. 2024 Jan 29;30(Suppl 1):1–7. doi: 10.1007/s44200-023-00041-5 (PMC11286687; doi:10.1007/s44200-023-00041-5)

**Supplementary material**

Tab. 1. Initial list of 118 terms from VascAgeNet network.

| augmentation index | Arterial tone | decompression wave |
| --- | --- | --- |
| characteristic impedance | calcification | digital biomarker |
| distensibility coefficient | vessell diameter | elastin |
| pulse wave velocity | impedance mis-match | frequency domain |
| wave separation analysis | input impedance | gold standard |
| compliance coefficient | oscillatory shear index | incident pressure wave |
| arterial stiffness | parameter | intimal calcification |
| arterial-ventricular coupling | Peterson’s modulus | longitudinal impedance |
| pulse pressure amplification | pulse pressure | measurement |
| wave intensity | pulse wave | mechanobiology |
| aortic blood pressure | reflection index | media calcification |
| endothelium | strain rate | medical imaging |
| reflection magnitude | systolic pressure amplification | model |
| wall shear stress | time averaged wall shear stress | muscular arteries |
| Young’s modulus | vascular calcifications | plaque |
| applanation tonometry | ventriculo-arterial coupling | precision |
| arterial age | vulnerable plaque | reflected pressure wave |
| arteriosclerosis | Ambulatory arterial stiffness index (AASI) | regulation of medical devices |
| augmentation pressure | cardio-ankle vascular index (CAVI) | repeatability |
| backward pressure wave | Large artery compliance | smooth muscle |
| elastic modulus | Small artery compliance | strain |
| forward pressure wave | Transfer function | strain rate |
| intima-media thickness | Wall shear stress gradient (WSSG) | steady state hemodynamics |
| pulsatile hemodynamics | accuracy | stress |
| pulse transit time | angiogenesis | technical validation |
| vascular ageing | arterial elongation | technology |
| Windkessel model | arterial growth / remodeling | terminal impedance |
| arterial elastance | arteriogenesis | time domain |
| atherosclerosis | arteriole | vasa vasorum |
| beta stiffness index | artery | vascular age |
| central blood pressure | atherogenesis | vascular remodeling |
| circumferential stress | biological age | vein |
| early vascular ageing | biomarker | volume elastic modulus |
| flow-mediated-dilation | blood flow rate | vascular growth |
| impedance | (24 hour) blood pressure variability | vasculogenesis |
| photoplethysmography | capillary | ventricular elastance |
| pressure augmentation | chronological age | wave |
| pulsatility index | clinical validation | wave speed |
| pulse arrival time | collagen |  |
| tonometry | compression wave |  |

Supplemental figure 1. Two examples of the published glossary terms: Compliance and Distensibility coefficients and Vulnerable Plaques.


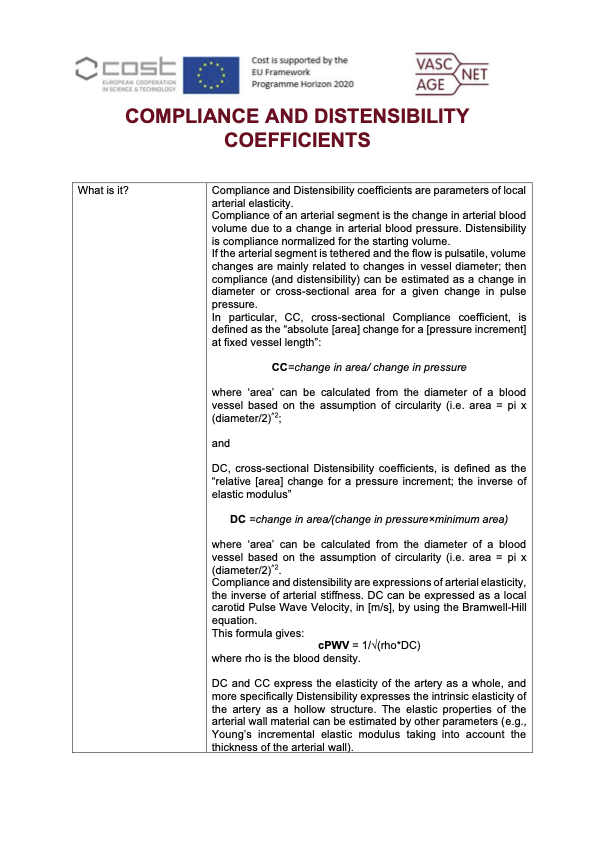


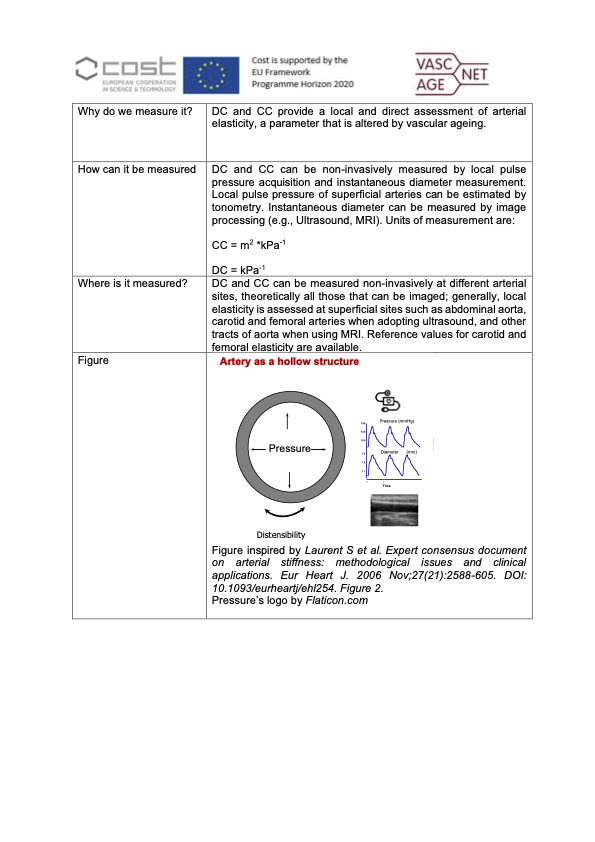


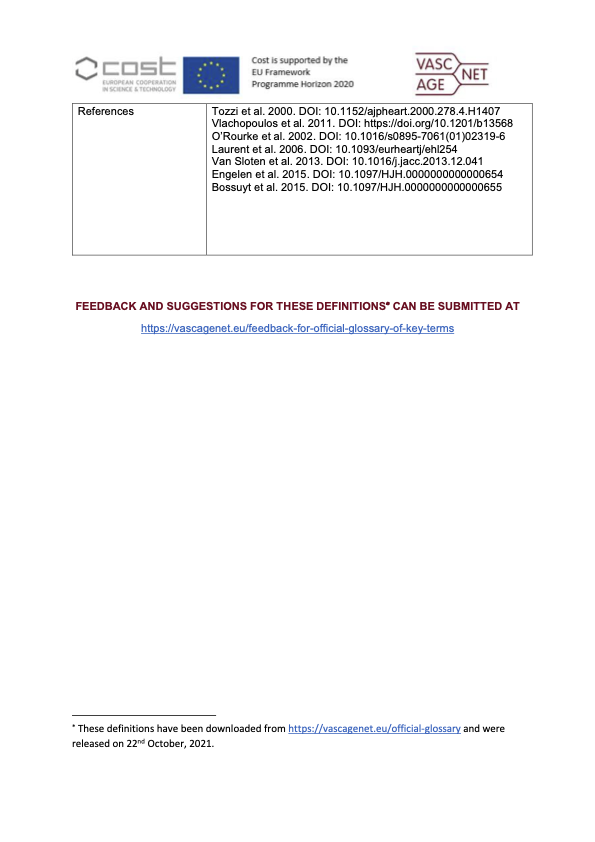


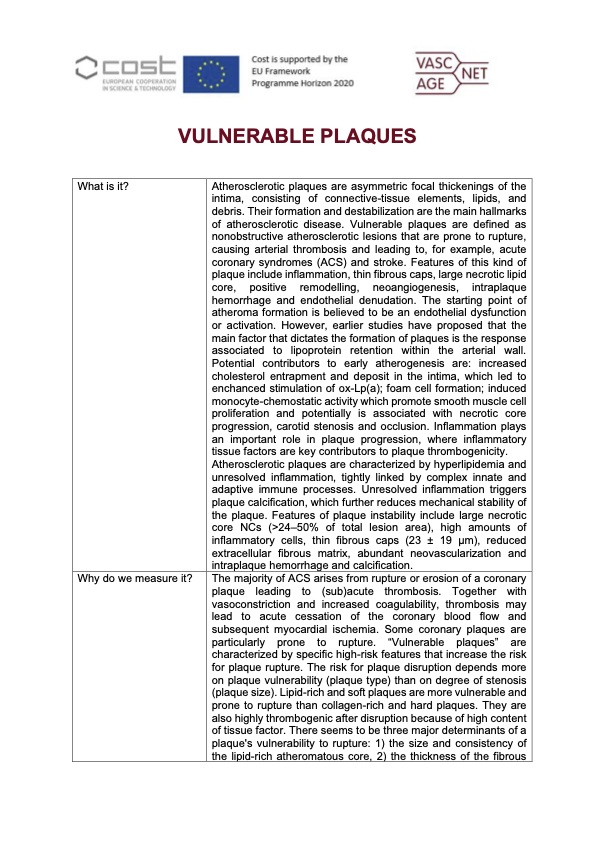

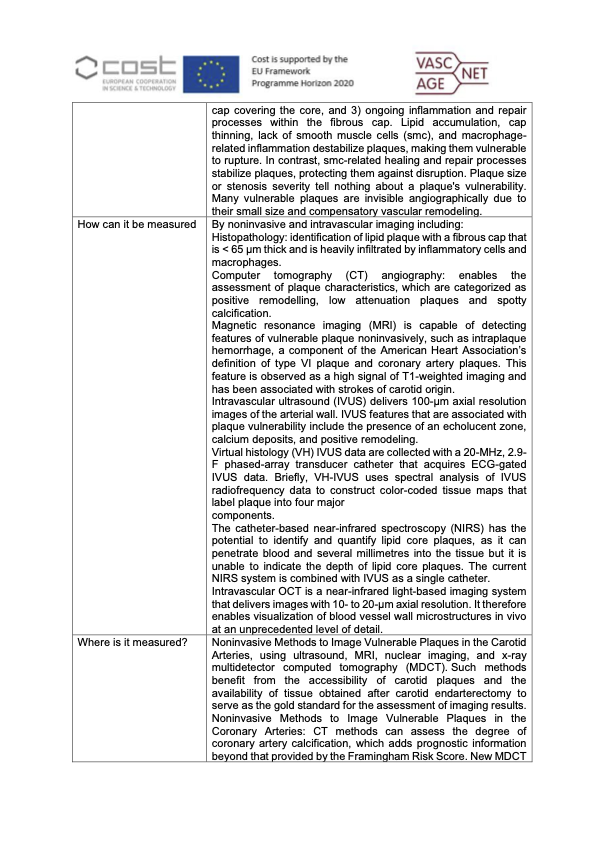

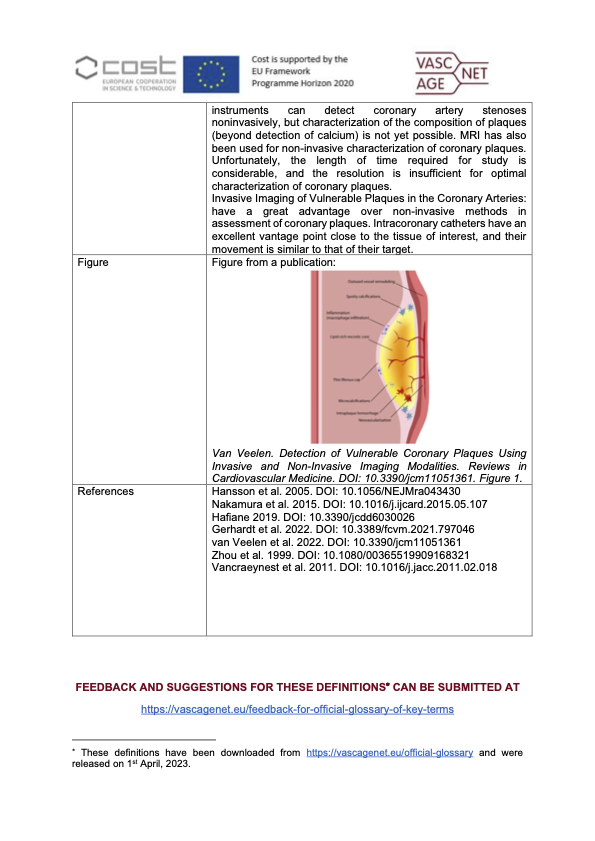

Supplement: Supplementary file 1 — Supplementary file1 (DOCX 1090 KB) [file 44200_2023_41_MOESM1_ESM.docx]
